# RNA Virus-Based Episomal Vector with a Fail-Safe Switch Facilitating Efficient Genetic Modification and Differentiation of iPSCs

**DOI:** 10.1016/j.omtm.2019.05.010

**Published:** 2019-05-28

**Authors:** Yumiko Komatsu, Dan Takeuchi, Tomoya Tokunaga, Hidetoshi Sakurai, Akiko Makino, Tomoyuki Honda, Yasuhiro Ikeda, Keizo Tomonaga

**Affiliations:** 1 Laboratory of RNA Viruses, Department of Virus Research, Institute for Frontier Life and Medical Sciences, Kyoto University, Kyoto 606-8507, Japan; 2 The Keihanshin Consortium for Fostering the Next Generation of Global Leaders in Research (K-CONNEX), Kyoto University, Kyoto 606-8501, Japan; 3 Section of Bacterial Drug Resistance Research, Thailand-Japan Research Collaboration Center, Research Institute for Microbial Diseases, Osaka University, Osaka 565-0871, Japan; 4 Center for iPS Cell Research and Application (CiRA), Kyoto University, Kyoto 606-8507, Japan; 5 Department of Mammalian Regulatory Network, Graduate School of Biostudies, Kyoto University, Kyoto 606-8507, Japan; 6 Division of Virology, Department of Microbiology and Immunology, Osaka University Graduate School of Medicine, Osaka 565-0871, Japan; 7 Department of Molecular Medicine, Mayo Clinic, Rochester, MN 55905, USA; 8 Department of Molecular Virology, Graduate School of Medicine, Kyoto University, Kyoto 606-8501, Japan

**Keywords:** episomal vector, RNA virus, iPSCs

## Abstract

A gene delivery system that allows efficient and safe stem cell modification is critical for next-generation stem cell therapies. An RNA virus-based episomal vector (REVec) is a gene transfer system developed based on Borna disease virus (BoDV), which facilitates persistent intranuclear RNA transgene delivery without integrating into the host genome. In this study, we analyzed susceptibility of human induced pluripotent stem cell (iPSC) lines from different somatic cell sources to REVec, along with commonly used viral vectors, and demonstrated highly efficient REVec transduction of iPSCs. Using REVec encoding myogenic transcription factor MyoD1, we further demonstrated potential application of the REVec system for inducing differentiation of iPSCs into skeletal muscle cells. Of note, treatment with a small molecule, T-705, completely eliminated REVec in persistently transduced cells. Thus, the REVec system offers a versatile toolbox for stable, integration-free iPSC modification and trans-differentiation, with a unique switch-off mechanism.

## Introduction

Gene transfer technology that allows efficient and stable genetic modification of induced pluripotent stem cells (iPSCs) is a key component in developing a successful gene therapy. To date, various viral and non-viral technologies have been used for gene transfer into iPSCs. Although the use of integrating viral vectors offer stable transgene expression in iPSCs, the risks of insertional mutagenesis and oncogenesis are recognized as major safety concerns surrounding these vectors.^1^ Non-integrating viral vectors provide transient transgene expression in proliferating cells and, therefore, may not be suitable for long-term expression in iPSCs.^2^, ^3^ Moreover, the efficiency of gene editing and off-target modifications represent major challenges in translating non-viral gene editing technologies for therapeutic applications.^4^

Another important feature for a safe gene delivery system is an ability to regulate transgene expression, or a suicide switch, as a fail-safe mechanism. Although several episomal vector systems are widely used in clinical applications, such as plasmid DNA, mini-circles, and adeno-associated virus (AAV) vectors,^5^ it has been highly challenging to accommodate a regulatable switch system or a self-degrading mechanism. For instance, there is no strategy currently available to completely eliminate AAV vector genomes in persistently transduced cells. Development of an episomal vector system with an integrated safety switch would pave a new path for safe gene therapy applications.

Borna disease virus (BoDV) is a single-stranded, negative-strand RNA virus that persistently infects a wide range of cell types.^6^ BoDV is unique among the other RNA viruses for its ability to replicate inside the nucleus without causing overt cytopathic effects.^7^ BoDV persists as episomes whereby viral ribonucleoprotein (vRNP) interacts with core histones that form the chromatin.^8^ During cell division, vRNPs are segregated along with host chromosomes into each of the daughter cell nuclei. Since vRNP persists as episomes, integration is not required for its intranuclear infection.^9^

We have previously exploited the unique characteristics of BoDV and developed a viral vector system.^10^, ^11^ We named this vector system an RNA virus-based episomal vector (REVec) system. In a follow-up study, we demonstrated that REVec achieves long-term transduction of human iPSCs without compromising its ability to differentiate into three embryonic germ layers.^12^ However, REVec transduction of mechanically dissociated iPSCs was relatively inefficient, and we were able to achieve up to 20% of iPSC transduction. In this study, we further explored potential applications of REVec for iPSC modification and transcription-factor-mediated induced differentiation. We demonstrated highly efficient (up to 80%) REVec transduction of single-cell-dissociated iPSCs with preserved pluripotency. REVec-mediated MyoD1 transfer induced iPSC differentiation into skeletal muscle cells. Importantly, treatment with T-705, a clinically approved small molecule, completely eliminated REVec genome in persistently transduced cells. Thus, our REVec system provides a unique genomic, modification-free, episomal gene delivery platform with a fail-safe switch-off system for stem cell modification.

## Results

### Susceptibility of Human iPSC Lines to REVec

**Figure 1:**
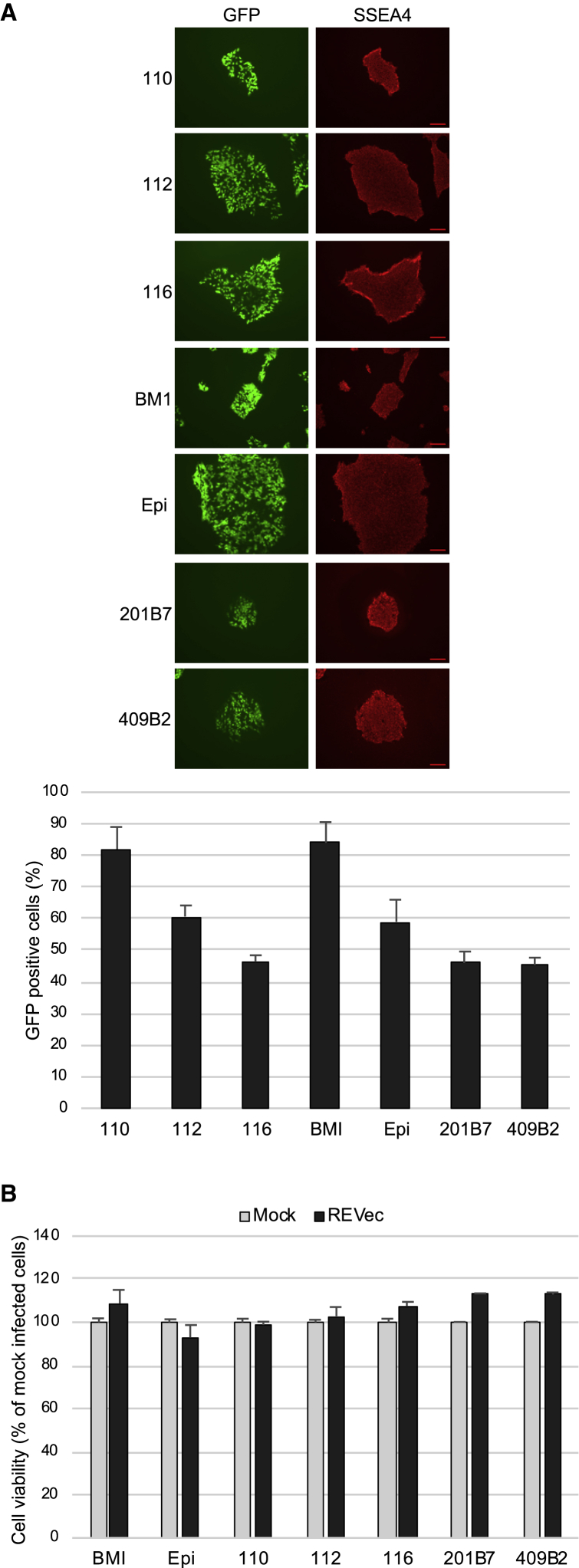
Susceptibility of iPSCs to REVec and Its Effect on Cell Viability (A) Human iPSCs (110, 112, 116, BMI, Epi, 201B7, and 409B2) were transduced with replication-defective REVec at a MOI of 1.0. At 1 week post-transduction, expression of GFP and pluripotency marker SSEA4 was analyzed (top panels). The percentage of GFP-positive cells was determined and reported as compared to mock (bottom panels). Scale bars, 100 μm. (B) Cell viability was determined by WST-1 assay at 1 week post-transduction and presented as percentage compared to mock. Data are shown as averages of two independent experiments with error bars represending SEM.

Previously, we reported sustained transgene expression of human iPSCs with REVec using a single iPSC line generated from primary dermal fibroblasts.^12^ To further investigate the efficiency of gene transfer into iPSCs derived from different somatic cell sources, seven human iPSC lines were plated on vitronectin coat as single cells and transduced with replication-defective REVec lacking the translation initiation codons for the glycoprotein gene^10^ at a MOI of 1.0. At 1 week post-transduction, expression of GFP and pluripotency marker SSEA4 were examined (Figure 1A). The percentage of GFP-positive cells ranged from 46.3% to 82% for bone-marrow-derived cells (110, 112, 116, and BMI), was 60.7% for cord-blood-derived cells (Epi [Gibco Episomal hiPSC line]), and ranged from 45.3% to 46% for fibroblast-derived cells (201B7 and 409B2). Expression of SSEA4 was confirmed in all transduced cells. To assess the cytotoxicity of REVec, cell viability was examined at 7 days post-transduction. As shown in Figure 1B, no significant change in cell viability was observed, indicating that REVec efficiently transduces iPSCs without appreciable cytotoxicity.

### Comparison of iPSC Transduction Efficiency by Various Viral Vectors

**Figure 2:**
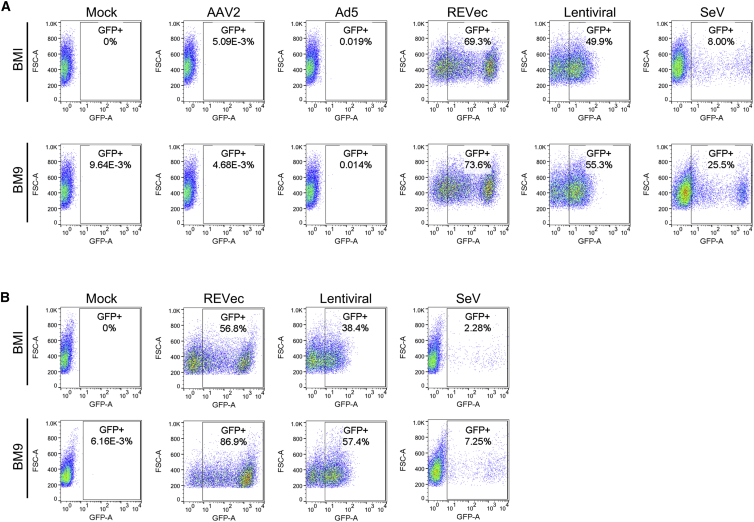
Efficiency of iPSC Transduction by Various Viral Vectors (A) BMI and BM9 iPSCs were transduced with AAV2, Ad5, REVec, lentiviral, and SeV vectors encoding GFP. At 1 week post-transduction, the percentage of GFP-positive cells was assessed by FACS analysis. (B) Percentage of GFP-positive cell populations at 2 weeks post-transduction with REVec, lentiviral, and SeV vectors.

To compare the efficiency of iPSC transduction by REVec and other commonly used viral vector platforms, iPSC lines BMI and BM9 were transduced with GFP-expressing AAV2 and adenovirus 5 (Ad5) vector at a MOI of 100 and with REVec, lentiviral, and Sendai virus (SeV) vector at a MOI of 10. The efficiency of gene delivery was determined at 1 and 2 weeks post-transduction by flow cytometry. At 1 week post-transduction, the percentage of GFP-positive cells was 69.3% for REVec, 49.9% for lentiviral vector, and 8.0% for SeV vector in BMI iPSCs (Figure 2A). The GFP-expressing cells were below the detection level in AAV2- and Ad5-vector-transduced cells. In BM9 iPSCs, the percentage of GFP-positive cells was highest with REVec (73.6%), followed by lentiviral vector (55.3%) and SeV vector (25.5%), and was below the detection limit for AAV2 and Ad5 vectors. To further examine the persistence of transgene expression, GFP-positive cell populations were next assessed at 2 weeks post-transduction (Figure 2B). Consistent with the result of 1 week, REVec achieved the highest efficiency of gene transfer into iPSCs.

### Differentiation Potentials of iPSCs Transduced with REVec and Lentiviral Vector

**Figure 3:**
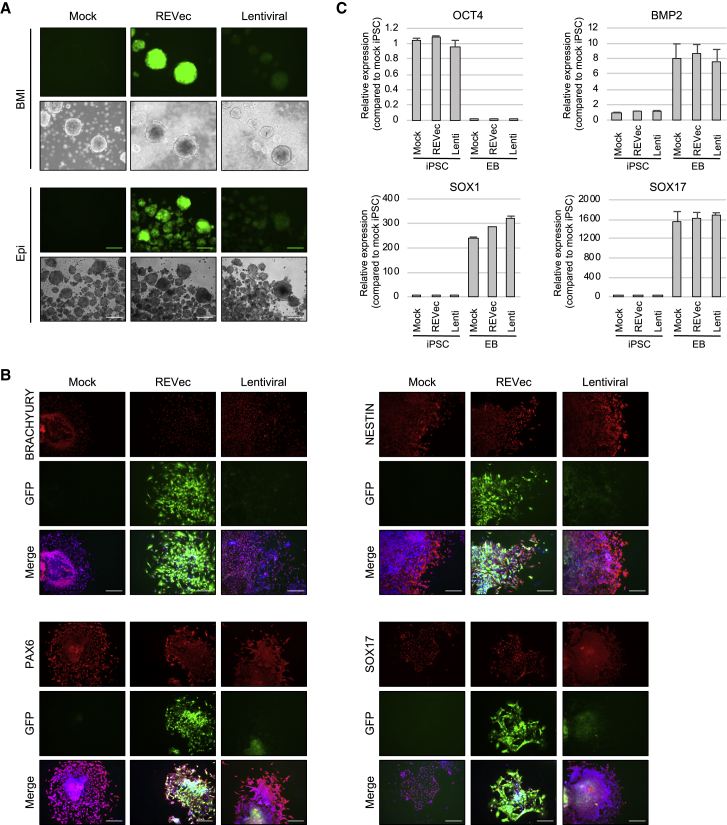
Differentiation Potentials of iPSCs Persistently Transduced with REVec and Lentiviral Vector (A) Embryoid bodies (EBs) were generated from BMI and Epi iPSCs transduced with REVec and lentiviral vector by culturing in suspension in DMEM containing 20% FCS. Scale bars, 100 μm. (B) EBs were dissociated into smaller clumps, plated on Matrigel-coated chamber slides, and analyzed by immunostaining with BRACHYURY, NESTIN, PAX6, and SOX17 antibodies. Cells were counterstained with DAPI. Scale bars, 100 μm. (C) Transcript levels of OCT4, BMP2, SOX1, and SOX17 in iPSCs and EBs were determined by real-time RT-PCR. The relative expression levels were normalized to β-actin and reported as compared to mock iPSCs. Data are shown as averages of three independent experiments with error bars representing SEM.

Since REVec and lentiviral vectors achieved >50% GFP-positive cells, differentiation potentials of iPSCs transduced with these vectors were next examined by embryoid body (EB) formation assay. BMI and Epi iPSCs persistently transduced with REVec or lentiviral vector both formed EBs when cultured in DMEM containing 20% serum (Figure 3A). Immunocytochemistry for three embryonic-germ-layer-specific genes—including BRACHYURY (mesoderm), NESTIN (ectoderm), PAX6 (ectoderm), and SOX17 (endoderm)—revealed no notable difference in the expression of these genes in mock-, REVec-, and lentiviral-vector-transduced cells (Figure 3B). Interestingly, while strong GFP expression was achieved by REVec, loss of GFP expression was observed in lentiviral-vector-transduced cells upon spontaneous differentiation. To further quantify these results, qRT-PCR analysis was conducted and showed a decrease in expression of the pluripotency marker (OCT4) and an increase in expression of the mesoderm marker (BMP2), ectoderm marker (SOX1), and endoderm marker (SOX17) in EBs, compared to that in iPSCs (Figure 3C). Together, these results indicate that non-integrating REVec achieves efficient gene transfer into iPSCs and that transgene expression is maintained upon differentiation.

### Myogenic Differentiation of iPSCs Using REVec Encoding MyoD1

**Figure 4:**
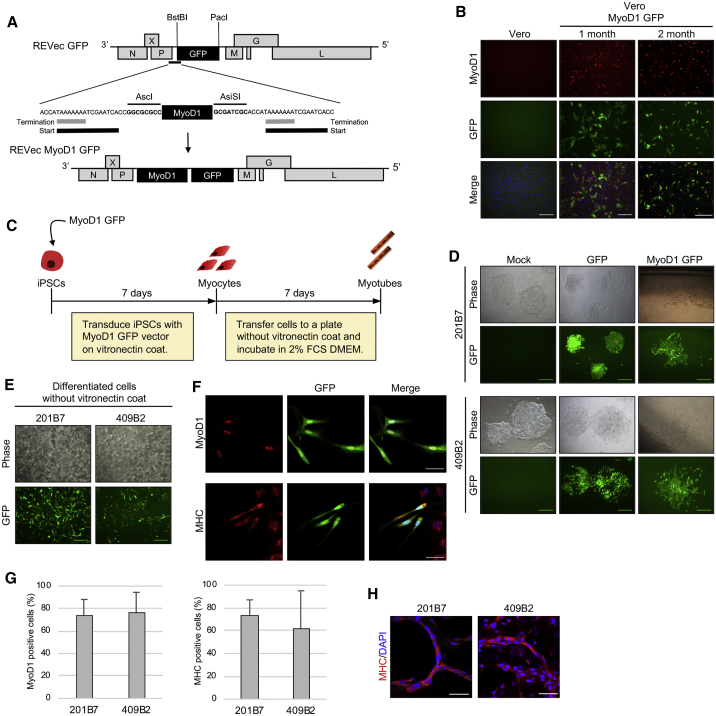
Myogenic Differentiation of iPSCs Using REVec (A) Genome organization of REVec encoding MyoD1 and GFP. (B) Expression of MyoD1 and GFP in vector-producing cells at 1 and 2 months post-transfection. Scale bars, 100 μm. (C) Schematic of myogenic differentiation of iPSCs using REVec. iPSCs were transduced with MyoD1 vector at a MOI of 1.0. Infected cells were cultured for 1 week in iPSC medium, followed by incubation in DMEM containing 2% FCS to further induce formation of myotubes. (D) 201B7 and 409B2 iPSCs were transduced with REVec-GFP or REVec-MyoD1 GFP. At 1 week post-transduction, GFP-positive cells were examined for differentiated morphology. Scale bars, 100 μm. (E) Differentiated cells after transfer to a plate without vitronectin coat. Scale bars, 100 μm. (F) Myogenic lineage of differentiated cells was assessed by immunostaining with MyoD1 and MHC antibodies. Scale bars, 50 μm. (G) Efficiency of myogenic differentiation by REVec. Ratio of MyoD1^+^DAPI^+^/DAPI^+^ cells and MHC^+^DAPI^+^/DAPI^+^ cells were determined and presented as percentage of MyoD1- and MHC-positive cells. Data are shown as averages of three independent experiments with error bars representing SEM. (H) One week after incubation in DMEM containing 2% FCS, multinucleated myotube formation was analyzed by immunostaining using MHC antibody and counterstaining with DAPI. Scale bars, 50 μm.

To exploit REVec for *in vitro* genetic modification of iPSCs, we next sought to develop a vector to induce differentiation of iPSCs into somatic cells. In order to achieve this, we used myogenic differentiation 1 (MyoD1), a transcription factor known as a master regulator of myogenesis, since overexpression of MyoD1 is sufficient to induce differentiation of iPSCs into skeletal muscle cells.^13^ Nevertheless, MyoD1 is not required once the cells are committed to myogenic lineage, thus providing us with a unique opportunity to test the small molecule inhibitor to switch off transgene expression from iPSC-derived cells. To generate REVec encoding MyoD1, we cloned MyoD1 cDNA from human rhabdomyosarcoma cell line TE671 in REVec vector plasmid containing GFP as a marker (Figure 4A). Vero cells stably expressing replication-competent REVec-MyoD1 GFP were generated by reverse genetics. The expression of MyoD1 and GFP was confirmed at 1 and 2 months post-transfection with vector plasmids, indicating generation of stable REVec-producing cells (Figure 4B).

To induce differentiation of iPSCs into skeletal muscle cells, cell-free vector was prepared from vector-producing cells, and 201B7 and 409B2 iPSCs were transduced with GFP or MyoD1 GFP vector (Figure 4C). At 1 week post-transduction, spindle-shaped myocyte-like cells were observed in both cell lines transduced with MyoD1 GFP vector (Figure 4D). In contrast, mock- and GFP-transduced cells maintained characteristic iPSC colony shape, indicating that the differentiated cells were specifically generated by forced expression of MyoD1 (Figure 4D). To further investigate whether these iPSCs have differentiated toward myogenic lineage, cells were next transferred to a plate without vitronectin coat to eliminate undifferentiated cells (Figure 4E) and analyzed for expression of myosin heavy chain markers, MyoD1, and myosin heavy chain (MHC) (Figure 4F). These cells were positive for MHC expression, indicating differentiation into myogenic lineage. To further quantify the efficiency of differentiation, the percentages of MyoD1- and MHC-positive cells were determined from the ratios of MyoD1^+^DAPI^+^/DAPI^+^ cells and MHC^+^DAPI^+^/DAPI^+^ cells (Figure 4G). The percentage of MyoD1-positive cells was 73% for 201B7 and 76% for 409B2 iPSCs, while the percentage of MHC-positive cells was 73% for 201B7 and 62% for 409B2 cells.

During skeletal muscle development, mononucleated myoblasts fuse to form multinucleated myotubes.^14^ Previous studies reported that incubation of myocytes in DMEM containing a low amount of serum induces the formation of myotubes.^15^ Therefore, differentiated cells were next cultured in DMEM supplemented with 2% FCS. After 1 week of incubation, multinucleated myotubes were generated (Figure 4H). Together, these results demonstrate the first example of an application of REVec for directed differentiation of iPSCs into a distinct cell lineage.

### Shutoff of REVec from Persistently Transduced Cells with T-705

**Figure 5:**
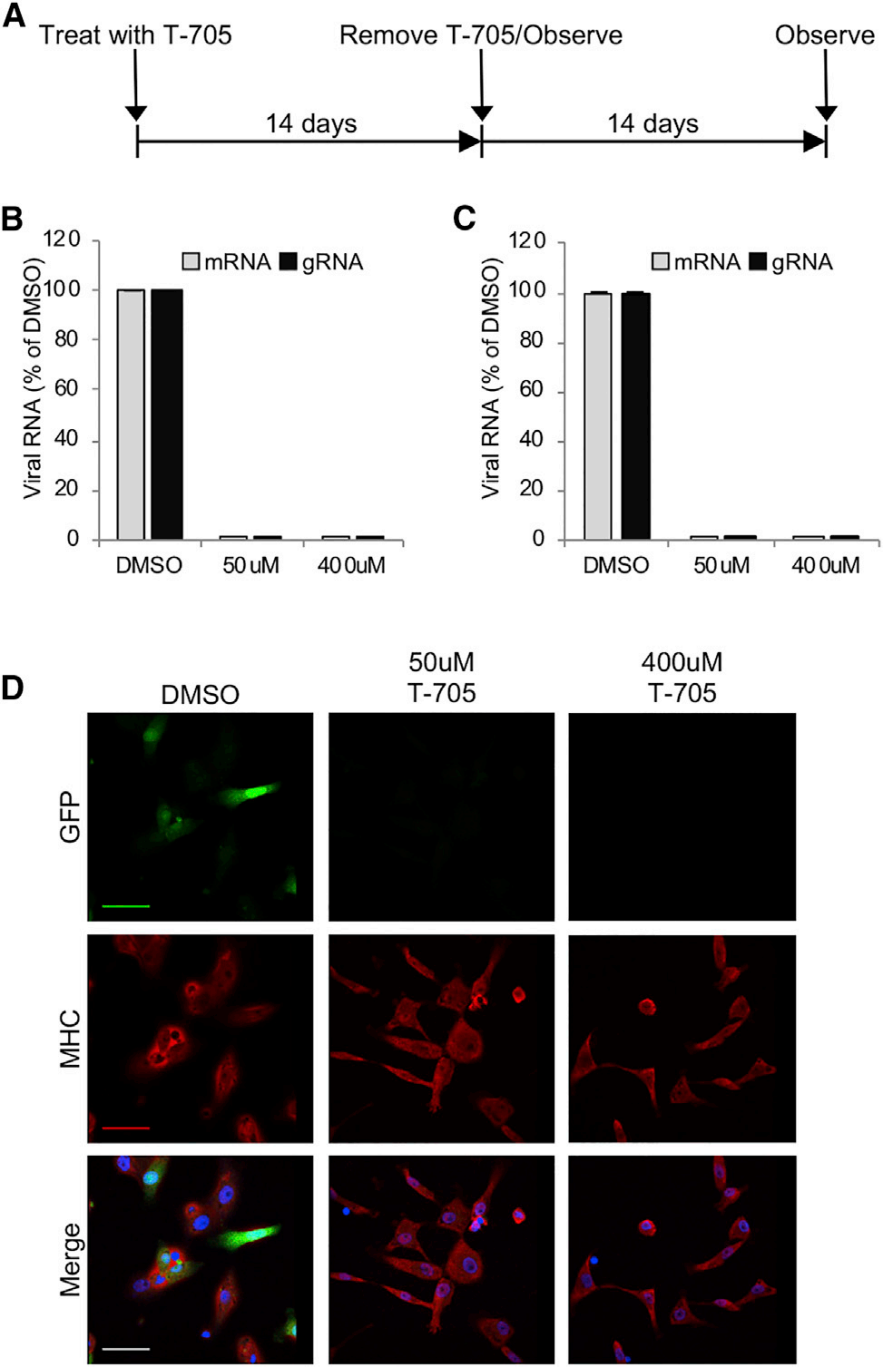
T-705 Eliminates REVec from iPSC-Derived Myocytes (A) Time course of T-705 treatment for removal of REVec from myocytes. (B) Real-time RT-PCR analysis for REVec mRNA and gRNA levels in myocytes after treatment with indicated concentrations of T-705 for 14 days. (C) REVec mRNA and gRNA levels at 14 days post-removal of T-705 from the culture medium. Data are shown as averages of three independent expreiments with error bars representing SEM. (D) Expression of GFP and MHC was assessed by immunostaining after removal of T-705 from culture medium. Cells were counterstained with DAPI. Scale bars, 50 μm.

A strategy that can turn off transgene expression from persistently transduced cells is crucial not only for regulating gene expression but also for ensuring the safety of the viral vector. Previously, we reported that nucleoside/nucleotide mimetics favipiravir (T-705) exerts an antiviral activity against mammalian bornavirus BoDV-1 strain He/80 and avian bornavirus PaBV-4.^16^ To examine whether transgene expression can be switched off, iPSC-derived myocytes were treated with 50 and 400 μM T-705, and mRNA and genomic RNA (gRNA) levels of REVec were assessed by real-time RT-PCR (Figure 5A). At 14 days post-treatment, mRNA and gRNA levels were below the detection limit by real-time RT-PCR (Figure 5B). To further confirm the absence of REVec, T-705 was removed from the medium, and the cells were cultured for an additional 14 days. As shown in Figure 5C, mRNA and gRNA levels of REVec remained below the detection limit. Furthermore, GFP protein expression remained undetectable after the removal of T-705 from culture medium (Figure 5D). Together, these results indicate that T-705 treatment can be used to turn off transgene expression from persistently transduced stem-cell-derived cells.

## Discussion

In the present study, we investigated the feasibility of using REVec for the genetic modification of iPSCs. Our results indicate that various human iPSC lines are highly permissive to REVec. A side-by-side comparison with commonly used viral vectors demonstrated highly efficient and persistent gene transfer into iPSCs and iPSC-derived cells upon differentiation by the non-integrating REVec system. Moreover, we demonstrated the first example of *in vitro* application of REVec for genetic modification of iPSCs by inducing differentiation into skeletal muscle cells by MyoD1 transfer and further showed shutoff of transgene expression from persistently infected cells using T-705.

Genetic modification tools that efficiently mediate long-term stable gene expression in iPSCs while maintaining its pluripotency are invaluable for stem cell research. The delivery of transgenes to iPSCs has an advantage over gene transfer into differentiated cells, as iPSCs can provide an unlimited source of somatic cells. Rapti et al. previously compared iPSC transduction efficiency by recombinant AAV2, AAV6, Ad5, and lentiviral vectors and reported that lentiviral and Ad5 vectors exhibit higher transduction efficiency than AAV2 and AAV6 vectors.^17^ In our study, REVec and lentiviral vectors transduced iPSCs more efficiently than Ad5 vector. This discrepancy could be attributed to differences in the iPSC line and viral vector constructs used in the present study.

Among the selected vectors examined, we observed cytotoxicity in SeV vector-transduced cells, which was marked by changes in cell morphology (data not shown). Since BoDV particles are rarely released into the supernatant, REVec, in the present study, was prepared by sonication to release cell-associated vector. This results in a large amount of cell-derived impurities in the vector stock that could potentially reduce its efficacy and induce cytotoxicity, depending on the target cell type. Nevertheless, we did not observe cytotoxicity by REVec when used at a MOI of 1 to 10 in iPSCs, whereas cellular toxicity could be induced at a higher MOI. Thus, we are currently developing a REVec purification strategy to improve its safety and efficacy. Gene delivery with lentiviral vector involves integration in the host genome that comes with a risk for insertional mutagenesis and oncogenic transformation. Development of lentiviral vector with reduced genotoxicity has been achieved by the use of self-inactivating (SIN) vectors with a synthetic chromatin insulator cassette;^18^ however, due to the possibility of clonal expansion of infected cells,^19^ the risk of mutagenesis is not completely eliminated. Moreover, transgenes expressed by lentiviral vector are prone to silencing due to CpG methylation in the promoter region and histone deacetylation.^20^ In contrast to integrating systems, REVec is based on an RNA virus with no viral DNA phase and persists in the cell by interacting with host chromosomes as an episomal RNA.^8^ Therefore, REVec is not prone to transgene silencing by an epigenetic mechanism, and the risk of genotoxicity remains markedly lower than that for the aforementioned system.

The packaging capacity of the vector system can substantially affect its downstream applications. In this study, REVec expressing MyoD1 (963 bp) and GFP (720 bp) in tandem (combined transgene size, 1,683 bp) was successfully rescued by reverse genetics. Additionally, REVec encoding larger transgenes Neprilysin (2,253 bp)^21^ and LacZ (3,060 bp)^10^ has been generated in our previous studies. Further investigation is required to determine its maximum packaging capacity.

REVec particles are rarely released into the supernatant and remain highly associated with vector-producing cells. Moreover, due to its non-cytolytic nature, once stable REVec-producing cells are generated, they can be cultured for a long-term for continuous vector production, as we have demonstrated in this study using Vero cells stably expressing MyoD1 and GFP. This presents an advantage over vector preparation by transient transfection method, as the stable vector-producing cells can be stored and expanded without the necessity to transfect cells prior to vector prep.

In this study, to demonstrate an application of REVec for genetic modification of iPSCs, a MyoD1-encoding vector was developed and used to induce differentiation of iPSCs into skeletal muscle cells. To date, various MyoD1 transfer systems have been used for the generation of skeletal muscle cells from iPSCs, including lentiviral vector expressing tamoxifen-inducible^22^ and doxycycline-inducible MyoD1,^23^ Ad5 expressing MyoD1,^15^ Tet-inducible MyoD1 piggyBac vector,^24^ and synthetic MyoD1 mRNA. In accordance with these studies, overexpression of MyoD1 by REVec generated MHC-positive myocytes. Although the purpose of this study was not to develop an efficient myogenic differentiation strategy, multinucleated myotubes were obtained in approximately 2 to 3 weeks using a two-step protocol, which was a relatively fast method compared to previously reported systems.

The ability to switch transgenes off from stably transduced cells is critical not only for controlling gene expression but also for limiting the potential adverse effects of therapy. However, currently, no strategy is available for removal of retroviral, lentiviral, and AAV vectors due to lack of antivirals and difficulty in eliminating integrated DNA. Remarkably, despite its ability to establish long-term persistence, REVec was eliminated with the clinically approved antiviral drug T-705. Recently, the persistent SeV vector, based on the noncytopathic persistent variant SeV Cl.151,^25^, ^26^ was developed. Notably, Nishimura et al.^26^ demonstrated that SeV Cl.151 can be removed from persistently infected HeLa cells with small interfering RNAs (siRNAs) targeting viral genes.^26^ Thus, persistent episomal RNA vector systems that can be switched off by small molecule inhibitor or siRNA will further enhance the safety of these long-term gene expression systems.

In conclusion, the present study demonstrates REVec as an effective and safe system for genetic modification of iPSCs. Although further studies are required to increase vector purity and to assess its safety *in vivo* before translating this vector into clinical applications, REVec presents a promising gene transfer system for *ex vivo* iPSC-based gene therapy, with a unique fail-safe strategy with T-705.

## Materials and Methods

### Cells and Chemicals

Vero cells were cultured in low-glucose DMEM (Nacalai Tesque, Kyoto, Japan) supplemented with 2% fetal calf serum (FCS). 293T cells were cultured in high-glucose DMEM (Thermo Fisher Scientific, Waltham, MA, USA) supplemented with 10% FCS. Human iPSCs 110 (BYS0110), 112 (BYS0112), and 116 (BYS0116), were obtained from ATCC (Manassas, VA, USA). BMI and BM9 were obtained from WiCell (Madison, WI, USA). 201B7 and 409B2 were obtained from the Riken Bioresource Center (Ibaraki, Japan). Epi was obtained from Thermo Fisher Scientific. Epi, BMI, BM9, 110, 112, and 116 were maintained on Corning Matrigel hESC-Qualified Matrix (CORNING, Corning, NY, USA) in mTeSR 1 medium (STEMCELL Technologies, Vancouver, BC, Canada). 201B7 and 409B2 were cultured on vitronectin (Thermo Fisher Scientific) in ReproFF2 medium (ReproCELL, Kanagawa, Japan) supplemented with 5 ng/mL basic fibroblast growth factor (bFGF; ReproCELL). T-705, Y27632, and puromycin were purchased from Selleckchem (Houston, TX, USA), FUJIFILM Wako Pure Chemical (Osaka, Japan), and InvivoGen (San Diego, CA, USA), respectively.

### Viral Vectors

EGFP-expressing REVec,^10^ SeV vector,^27^ AAV2 vector,^28^ Ad5 vector,^29^ and HIV-SFFV-EGFP vector^30^ were described previously. To generate replication-competent REVec expressing MyoD1 and EGFP, MyoD1 was PCR amplified from TE671 cells and inserted into the pFct-BoDV P/M-EGFP plasmid. Recombinant REVec was rescued by reverse genetics, as described previously.^10^ Briefly, 293T cells were co-transfected with pFct-BoDV P/M-MyoD1 EGFP and helper plasmids (N, P, and L) using Lipofectamine 2000 (Thermo Fisher Scientific) and overlaid with puromycin-resistant Vero cells. At 5 days post co-culture, cells were treated with puromycin and passaged until the majority of the cells became positive for vector production.

To determine the titers of EGFP-expressing lentiviral and SeV vectors, a total of 2 × 10^5^ 293T cells per well in 24-well plates were inoculated with serial dilutions of the vector supernatants overnight. Numbers of infected cells were determined by measurement of EGFP expression by fluorescence-activated cell sorting (FACS) using a FACScan and CELL QUEST software (Becton Dickinson, Franklin Lake, NJ, USA), and vector titers (infectious units [IUs] per milliliter) were estimated as follows: (2 × 10^5^) × (% EGFP positive/100) × (1,000/microliters of infected viral vectors). The titers (genomic copy numbers per milliliter) of iodexanol-gradient-concentrated AAV and CsCl banding-purified adenoviral vector stocks were determined by real-time PCR using plasmid DNA standards and AAV genomic sequence-specific primers. The titer of REVec was determined by inoculating 2 × 10^4^ 293T cells seeded in a 96-well plate with serial dilutions of vector prep obtained by sonication of vector-producing cells. After absorption at 37°C for 2 h, the cells were washed and replaced with fresh medium and incubated for 72 h. Vector titer was determined by counting the number of EGFP-positive cells using a fluorescence microscope.

### Transduction of iPSCs with REVec and Measurement of Cytotoxicity

iPSCs were dissociated into a single-cell suspension using ESGRO Complete Accutase (Merck Millipore, Burlington, MA, USA) and seeded in a 12-well plate at 0.3 × 10^4^ cells per well. Rock inhibitor Y27632 was added at a final concentration of 10 μM. On the next day, cells were replaced with medium without Y27632 and inoculated with REVec at a MOI of 1.0 in a total volume of 200 μL. After absorption for 3 h at 37°C in a 5% CO_2_ incubator, supernatant containing the vector was replaced with fresh iPSC medium. The efficiency of iPSC transduction was determined by flow cytometry (BD Biosciences, San Jose, CA, USA) and the Tali Image-Based Cytometer (Thermo Fisher Scientific). The cytotoxicity of REVec was determined by WST-1 assay according to the manufacturer’s instructions (Takara Bio, Shiga, Japan).

### Differentiation of iPSCs into Skeletal Muscle Cells

201B7 and 409B2 iPSCs were seeded in a vitronectin-coated 12-well plate at 0.3 × 10^4^ cells per well and transduced with REVec-MyoD1 EGFP at a MOI of 1.0. At 1 week post-transduction, differentiated cells were transferred to a fresh 12-well plate without vitronectin coat and incubated in DMEM supplemented with 2% FCS for 1 week to induce formation of myotubes.

### Immunostaining

The cells were fixed with 4% paraformaldehyde, permealized in 0.4% Triton-X, and blocked with PBS containing 2% BSA, followed by incubation with the following primary antibodies: BRACHYURY monoclonal antibody X1AO2 (eBioscience, San Diego, CA, USA), Anti-SOX17 (R&D Systems, Minneapolis, MN, USA), PAX6 polyclonal antibody (Thermo Fisher Scientific), NESTIN monoclonal antibody 10C2 (Thermo Fisher Scientific), MyoD1 D8G3 (Cell Signaling Technology, Danvers, MA, USA), and anti-myosin heavy chain (R&D). After 1 h of incubation with primary antibody, cells were next washed with PBS and incubated with the following secondary antibodies: goat anti-rabbit IgG (immunoglobulin G) conjugated with Alexa Fluor 555, donkey anti-goat IgG conjugated with Alexa Fluor 555, or goat anti-mouse IgG conjugated with Alexa Fluor 555, for 1 h at room temperature. Cells were next washed with PBS, and counterstained with DAPI. Secondary antibodies and DAPI were obtained from Thermo Fisher Scientific. Fluorescence images were taken with an Eclipse TE2000-U inverted microscope (Nikon, Shinagawa, Japan) or with a Ti-E inverted microscope with a C1 confocal laser scanning system (Nikon).

### EB Formation Assay

EBs were formed as previously described.^12^ Briefly, iPSCs were dissociated into single-cell suspension and cultured on low-adhesion plates in mTESR 1 medium for 4 days, followed by incubation in DMEM supplemented with 20% FCS for 1 week. For immunostaining, EBs were dissociated into smaller clumps and plated on Matrigel-coated chamber slides.

### Real-Time RT-PCR

Total RNA was isolated from iPSCs and EBs using TRIzol Reagent (Thermo Fisher Scientific), and cDNA was synthesized using the RevertAid First Strand cDNA Synthesis Kit (Thermo Fisher Scientific), using oligo(dT)_18_ primer. qPCR analysis was performed using the Power SYBR Green PCR Master Mix (Applied Biosystems, Waltham, MA, USA) and analyzed on the StepOnePlus Real-Time PCR System (Applied Biosystems). The cycling conditions were as per the manufacturer’s instructions: 95°C for 10 min followed by 40 cycles of 95°C for 15 s and 60°C for 1 min. The following primers were used: OCT4 (5′-gaaggatgtggtccgagtgt-3′ and 5′-gcctcaaaatcctctcgttg-3′), BMP2 (5′-tgtgtcccgacagaactcag-3′ and 5′-acaaccctccacaaccatgt-3′), SOX1 (5′-ctgacaccagacttgggttt-3′ and 5′-aagaaaacgctttccgcttcc-3′), SOX17 (5′-atgggcggagttatgatacctac-3′ and 5′-attcacaccggagtcatgc-3′), and β-actin (5′- ggcatcctcaccctgaagta-3′ and 5′-aggtgtggtgccagattttc-3′). The levels of REVec mRNA and gRNA were analyzed as described previously,^16^ using the following primers: oligo(dT) primer or BoDV-1 genome-specific primer (5′-tgttgcgctaacaacaaaccaatcac-3′) for cDNA synthesis and BoDV-1 P primers (5′-atgcattgacccaaccggta-3′ and 5′-atcattcgatagctgctcccttc-3′) for quantification of RNA.

## Author Contributions

Conceptualization, Y.K., D.T., and K.T.; Investigation, Y.K., D.T., and T.T.; Resources, Y.I., A.M., and H.S.; Writing – Original Draft, Y.K.; Writing – Review & Editing, Y.K., K.T., Y.I., and T.H.; Supervision T.K.; Funding Acquisition, Y.K. and K.T.

## Conflicts of Interest

The authors declare no competing interests.
